# Associations of Skin Autofluorescence with Diabetic Kidney Disease in Type 2 Diabetes

**DOI:** 10.3390/biomedicines13040764

**Published:** 2025-03-21

**Authors:** Ziwei Liu, Jingjie Wang, Yuedong Zhao, Zhu Yuan, Xinjuan Zhuang, Jun Yin

**Affiliations:** 1 Department of Endocrinology and Metabolism, Jinshan Branch of Shanghai Sixth People’s Hospital, Shanghai 201599, China; 15584382922@163.com (Z.L.); zhaoyuedong0531@163.com (Y.Z.); 15972569315@163.com (Z.Y.); 2 Shanghai Key Laboratory of Diabetes Mellitus, Shanghai Diabetes Institute, Shanghai Clinical Center for Diabetes, Department of Endocrinology and Metabolism, Shanghai Sixth People’s Hospital Affiliated to Shanghai Jiao Tong University School of Medicine, Shanghai 200233, China; jingjiewang_med@163.com

**Keywords:** diabetic kidney disease, type 2 diabetes mellitus, advanced glycation end products, renal function

## Abstract

**Background**: Diabetic kidney disease (DKD), a severe chronic complication of diabetes, significantly impacts the quality of life and life expectancy of affected individuals. Meanwhile, advanced glycation end products (AGEs) are believed to play a central role in the pathogenesis of DKD. Skin autofluorescence (SAF) is a well-validated, noninvasive technique for the estimation of AGE levels in the dermis. **Aims**: This study aims to evaluate the correlation between SAF and DKD prevalence, as well as the association between SAF and renal function parameters, in patients with Type 2 Diabetes Mellitus (T2DM). **Methods**: This cross-sectional analysis included 1259 hospitalized T2DM patients. SAF was measured using a spectroscopy device. Logistic regression analysis, *p*-trend analysis, and restricted cubic spline were performed with the prevalence of DKD as the dependent variable. Multiple linear regression analyses were conducted to investigate the associations of SAF with renal function parameters, specifically the estimated glomerular filtration rate (eGFR) and the log-transformed albumin-to-creatinine ratio (ln(ACR)). **Results**: The prevalence of DKD was strongly associated with SAF rather than with glycosylated hemoglobin (HbA1c). For each arbitrary unit (AU) increase in SAF, DKD incidence rose by 1.6%. A significant stepwise increase in the odds ratio (OR) of DKD was observed across SAF quartiles. A dose-response relationship existed between SAF and the OR value of DKD. Additionally, SAF showed a linear correlation with eGFR and ln(UACR). For each AU increase in SAF, eGFR decreased by 0.14 mL/min/1.73 m^2^, while UACR increased by 1.2%. **Conclusions**: Elevated SAF, rather than HbA1c, is independently associated with increased DKD prevalence and impaired renal function.

## 1. Introduction

The global prevalence of diabetes mellitus, particularly type 2 diabetes mellitus (T2DM), continues to rise at an alarming rate, prompting the World Health Organization (WHO) to classify T2DM as a significant public health threat [1,2]. Diabetic kidney disease (DKD) develops in approximately 40% of T2DM patients [3]. It is the leading cause of end-stage kidney disease (ESKD), which is associated with increased cardiovascular and all-cause mortality [4,5]. Despite advancements in risk factor control and the introduction of novel treatments such as sodium-glucose co-transporter-2 (SGLT2) inhibitors, the prevalence of DKD remains concerningly high [3]. This underscores the urgent need for a reliable prognostic marker to identify T2DM patients at high risk for DKD.

Advanced glycation end products (AGEs) are a group of products generated nonenzymatically and irreversibly from the reactions between sugars and macromolecules (proteins, lipids, and nucleic acids) [6]. AGEs form and accumulate in the human body during aging, and diabetes has been confirmed to accelerate this process, leading to increased AGE deposition in tissues and consequent pathological conditions [7]. In 1986, Monnier et al. first reported the association between the accumulation of AGEs in skin tissue and the presence of vascular complications in patients with type 1 diabetes mellitus (T1DM) [8]. Subsequent research revealed that both T1DM and T2DM patients with ESKD exhibit significantly higher tissue AGE levels compared to those without renal disease [9].

In the early days, clinical application of AGEs accumulation measurement in diabetes and its complications was relatively limited due to the invasive nature of tissue biopsies. Nowadays, the development of skin autofluorescence (SAF), a noninvasive technique, has enabled a more accessible evaluation of AGE accumulation, sparking researchers’ interest in its potential use for screening and predicting DKD and other diabetes-related complications. In the meantime, though albuminuria remains a classic presentation and diagnostic criterion of DKD, recent studies suggest that certain DKD patients exhibit reduced renal function without albuminuria, accompanied by prominent vascular and interstitial fibrosis on renal histology [10]. Hence, it is essential to further evaluate the association between SAF level and DKD as skin SAF may provide more comprehensive information than some traditional parameters.

In this study, we elucidated the detailed association between SAF and DKD prevalence. Additionally, we evaluated the relationship between SAF and parameters of renal function in patients with T2DM.

## 2. Methods

### 2.1. Study Population

This cross-sectional study was conducted in the Department of Endocrinology and Metabolism, Jinshan Branch of Shanghai Sixth People’s Hospital. Hospitalized patients with T2DM between December 2021 and April 2023 were selected and included. T2DM diagnoses were made based on the 2012 American Diabetes Association guidelines [1]. Patients with any of the following conditions were excluded: (1) type 1 or special types of diabetes, (2) acute complications of diabetes, (3) any febrile or infectious illness, (4) severe heart failure or stroke, (5) liver diseases or renal dysfunction, (6) malignant tumors, (7) autoimmune diseases or pharmacological treatment within three months, and (8) pregnant women. A total of 1259 patients with T2DM were eventually enrolled.

### 2.2. Definition and GA-Classification of DKD

In this study, the definition and classification of DKD stages were based on the KDIGO criteria [11]. DKD was defined as a UACR ≥ 30 mg/g and/or an eGFR < 60 mL/min/1.73 m^2^. Stages of DKD were classified based on two parameters, UACR and eGFR. Based on UACR, DKD was categorized into three stages: A1 (UACR < 30 mg/g, normal), A2 (30 mg/g ≤ UACR < 300 mg/g, microalbuminuria), and A3 (UACR ≥ 300 mg/g, macroalbuminuria). Based on eGFR, DKD was classified into five stages: G1 (eGFR ≥ 90 mL/min/1.73 m^2^), G2 (eGFR = 60–89 mL/min/1.73 m^2^), G3 (eGFR = 30–59 mL/min/1.73 m^2^), G4 (eGFR = 15–29 mL/min/1.73 m^2^), and G5 (eGFR < 15 mL/min/1.73 m^2^).

### 2.3. Assessment of Clinical Parameters

Data on demographics, medical history, medication, and laboratory indicators were collected from the hospital’s electronic data capture system. Clinical parameters potentially associated with the progression of T2DM and/or DKD were extracted as comprehensively as possible. BMI was calculated as weight (kg) divided by the square of height in meters (m^2^). Blood pressure was measured on the right arm in a sitting position three times consecutively at 5-minute intervals, with the mean value used for analysis. Current smokers were defined as individuals who had smoked at least 100 cigarettes in their lifetime and had not quit by the index date. Hypertension was defined as a systolic blood pressure ≥ 140 mmHg and/or a diastolic blood pressure ≥ 90 mmHg on at least two separate occasions, regardless of antihypertensive treatment. Blood samples were collected the morning after hospital admission, with patients fasting for at least 12 h before collection. Biochemical parameters, including glycated hemoglobin A1c (HbA1c), glycated albumin (GA), fasting plasma glucose (FPG), fasting C-peptide (FCP), serum lipids, and renal function, were assayed as previously reported [12]. In particular, HbA1c was measured via high-performance liquid chromatography (HPLC) with a VARIANT II Hemoglobin A1c analyzer (BioRad Laboratories, Hercules, CA, USA).

### 2.4. Assessment of SAF Levels

SAF levels were measured using a spectroscopy device developed by the Hefei Institutes of Physical Science, Chinese Academy of Sciences, Anhui Province, China. The device consists of an ultraviolet light source, a broadband light source, a trifurcated fiber-optic probe, and a compact charge-coupled device spectrometer [13]. Excitation light with a peak wavelength of 370 nm was used to stimulate AGEs in the skin, which emit fluorescence within a 420–600 nm wavelength range. Skin diffuse reflectance at 350–600 nm was also measured to correct for tissue absorption and scattering. The SAF value, indicative of skin AGEs accumulation, is determined through an integrative analysis of fluorescence and diffuse reflectance data using a built-in algorithm [14]. Measurements were performed by trained nurses at room temperature in a semi-dark environment. The volar side of the left arm was measured three times, and the mean value was calculated for analysis.

### 2.5. Statistical Analysis

Analyses were conducted using the R software, version 4.4.1. Data normality was assessed using the Shapiro–Wilk test and quantile-quantile (Q-Q) plots. Statistical evaluations of baseline characteristics between/among groups were conducted through independent samples *t*-test or analysis of variance (ANOVA) when the variable was normally distributed or the Mann–Whitney Wilcoxon test when a nonnormality was suggested. Chi-square tests were also conducted to compare the frequencies of categorical variables. Propensity score matching (PSM) was applied to match patients with and without DKD at a 1:1 ratio, using age, T2DM duration, HbA1c, systolic blood pressure (SBP), and diastolic blood pressure (DBP) as matching variables. The caliper value was set to 0.2. Logistic regression and multiple linear regression were performed to relate SAF, along with other potential covariates, to DKD prevalence and renal function parameters (eGFR and log-transformed UACR). To further characterize the association between SAF and the odds of developing DKD, *p*-trend analysis based on SAF quartiles and restricted cubic spline (RCS) regression analysis were performed. The *p*-trend analysis included an unadjusted regression model (model 1) and a model adjusted for confounding factors (model 2), including age, C-peptide, the duration of T2DM, triglyceride (TG), high-density lipoprotein cholesterol (HDL-C), hypertension history, and serum uric acid (SUA). The RCS analysis was conducted on all participants and matched participants to evaluate potential confounding effects. All statistical tests in this study were two-tailed, and differences were considered significant at a *p*-value < 0.05.

## 3. Results

A total of 1259 patients with T2DM were included in the final analysis. The baseline characteristics of the participants are summarized in Table 1. The study subjects were classified into two groups based on the presence or absence of DKD: the non-DKD group (682 patients) and the DKD group (577 patients). A comparison of the baseline characteristics between the two groups revealed that SAF levels were significantly higher in subjects with DKD, while the mean (±SD) value of HbA1c showed no prominent statistical difference between the two groups. Patients with DKD were also slightly older in age and had a longer duration of diabetes. They had elevated levels of C-peptide, TG, UACR, and SUA, as well as lower levels of HDL-C and eGFR. Additionally, they were more likely to have a history of hypertension, use antihypertensive medications, and undergo insulin therapy.

All participants were further stratified based on the UACR levels and eGFR levels. When stratified by UACR values, subjects were allocated into stages A1 (726 patients), A2 (360 patients), and A3 (173 patients). Significant differences (*p* < 0.05) were observed across the three groups in terms of the duration of diabetes, C-peptide, HDL-C, eGFR, UACR, SAF, SUA, and the use of antihypertensive medications.

When stratified by eGFR levels, the patients were classified into stages G1 and G2 (1119 patients), G3 (114 patients), and G4 and G5 (26 patients). Significant differences (*p* < 0.05) were identified among the three groups in terms of age, the duration of diabetes, HbA1c, C-peptide, GA, TG, HDL-C, eGFR, UACR, SAF, SUA, hypertension history, current smoking condition, the use of antihypertensive medications, and insulin injection.

PSM analysis was done to create a subgroup of matched participants, aiming to control for confounding factors in the RCS regression analysis. Patients were matched in a 1:1 case-control design based on age, T2DM duration, SBP, and DBP, as age is closely related to SAF levels [15], and both T2DM duration and blood pressure are verified risk factors for DKD [16,17]. It should be noted that after controlling the confounding factors, the DKD group still exhibited a significantly higher level of SAF than non-DKD patients (*p* < 0.001) (Table 2).

### 3.1. Logistic Regression Analysis Between SAF and DKD Incidence

Logistic Regression Analyses were performed to examine the independent association of SAF with DKD prevalence (Table 3). The results from simple logistic regression analysis demonstrated that SAF, as well as the duration of diabetes, C-peptide, HDL-C, and SUA, were significant risk factors for DKD. These variables were then selected as covariates for stepwise multiple regression. In stepwise multiple logistic regression, SAF, the duration of diabetes, C-peptide, and SUA still showed significance, while HDL-C was no longer a significant risk factor for DKD. The results from both simple and multiple logistic regression analysis demonstrated that SAF tends to have a strong association with the prevalence of DKD. Each arbitrary unit (AU) increase in SAF was associated with a 1.6% increase in DKD prevalence, as indicated by the OR value of stepwise multiple regression analysis.

### 3.2. Linear Regression Analysis Between SAF and Renal Function Parameters

**Figure 1:**
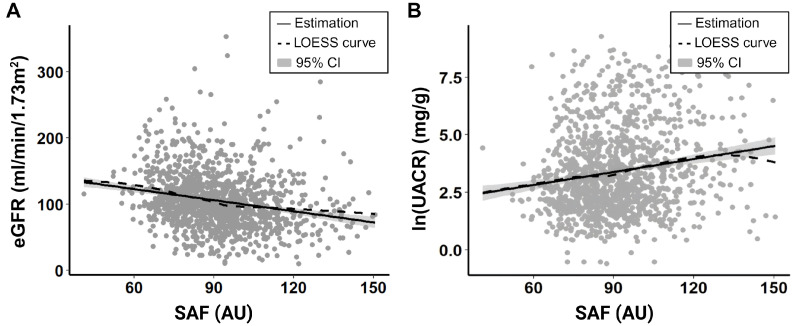
Association between SAF and renal function parameters. (**A**) eGFR, (**B**) log-transformed UACR.

Table 4 and Table 5 and Figure 1 summarize two multiple linear regression analyses we performed to evaluate whether there was any correlation between SAF levels and renal function parameters. Results of the two linear regression analyses showed that SAF was negatively correlated with eGFR (β = −0.140, *p* = 0.020) and positively correlated with log-transformed UACR (ln(ACR)) (β = 0.012, *p* < 0.001). Both correlations were approximately linear as the LOESS curves in Figure 1A,B closely followed their corresponding linear trend line. For every 1 AU increase in SAF, the eGFR of T2DM patients decreased by 0.14 mL/min/1.73 m^2^, while UACR increased by 1.2%.

In addition to SAF, some other variables also showed associations with renal function in T2DM patients. Age, C-peptide, SUA, and a hypertension history negatively correlated with eGFR, as demonstrated in Table 4. Duration of T2DM, C-peptide, and SUA positively correlated with ln(ACR), as demonstrated in Table 5.

### 3.3. Distribution of Renal Function Stages in T2DM Patients with Different SAF Levels

**Figure 2:**
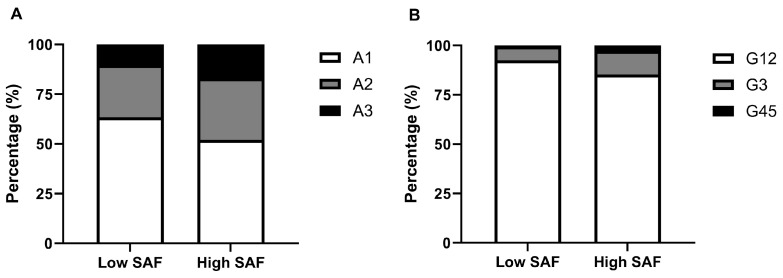
The composition of renal function stages in T2DM patients with different SAF levels. (**A**) eGFR-based stages, (**B**) UACR-based stages.

To explore the distribution of eGFR-based and UACR-based renal function stages in T2DM patients with different SAF levels, we divided all participants into two groups; patients with SAF levels lower than 89.3 AU (the median SAF value of all subjects) were classified as the low SAF group, while patients with SAF levels of 89.3 AU or higher were classified as the high SAF group. The results of the chi-square test revealed that the DKD incidence rate of the high SAF group was significantly higher than that of the low SAF group (52.1% vs. 39.5%). Figure 2A shows that the proportion of patients at stage A2 and stage A3 were both higher in the high SAF group when compared with the low SAF group (30.9% vs. 26.2.%, 17.0% vs. 10.4%). Similarly, Figure 2B demonstrates that in the high SAF group, patients at stage G1 and G2 accounted for a smaller percentage than in the low SAF group (85.3% vs. 92.5%), while patients at stage G3 or stage G4 and G5 made up a much larger proportion in relative to the low SAF group (11.5% vs. 6.6%, 3.1% vs. 1.0%).

### 3.4. Relationship of the Quartiles of SAF Levels with the OR of DKD in All Participants

**Figure 3:**
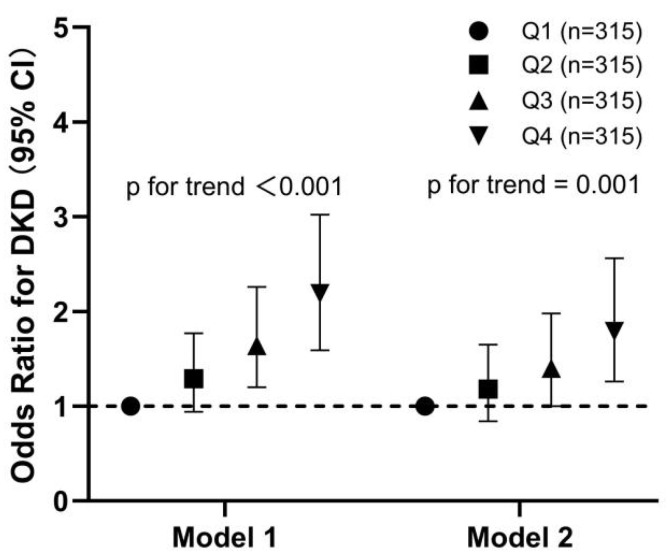
Relationship of the quartiles of SAF with the incidence of DKD in all participants. Model 1, unadjusted model. Model 2, adjusted for age, C-peptide, duration of T2DM, TG, HDL-C, SUA, and hypertension history. Error bars indicate 95% CI.

To further evaluate the relationship between SAF and DKD, all participants were stratified into four groups based on the quartiles of SAF levels (Figure 3). A significant stepwise increase in OR of DKD across quartiles of SAF was observed in the unadjusted regression model (*p* < 0.001). Adjustment for confounding factors (age, C-peptide, duration of T2DM, TG, HDLC, hypertension history, and SUA) slightly attenuated the strength of this association, but the trend remained strongly significant (*p* = 0.001).

### 3.5. RCS Regression Analysis Examining the Association Between SAF Levels and the Occurrence of DKD

**Figure 4:**
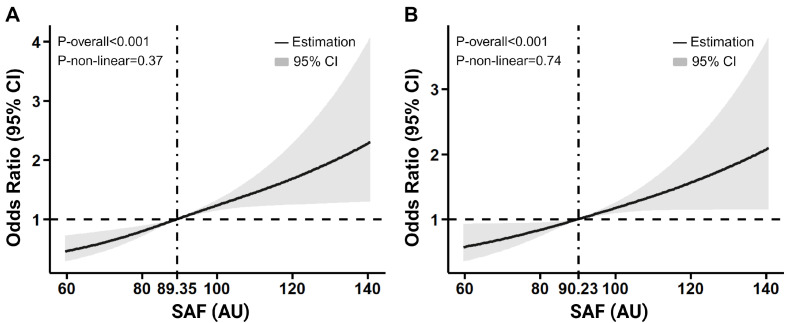
Dose-response associations between SAF and OR of incident DKD in all participants and matched participants. (**A**) all participants, (**B**) participants matched by age, duration, HbA1c, SBP, and DBP. The horizontal dashed line denotes an odds ratio of 1 (no association). The vertical dotted line represents the SAF level at which OR = 1 (reference SAF level).

To better understand the dose-response relationship between SAF and the odds of developing DKD in T2DM patients, restricted cubic spline (RCS) regression analyses were performed in all participants and matched participants, respectively (Figure 4). The results revealed a linear pattern, indicating that higher SAF levels were associated with increased DKD odds (*p* < 0.001). This relationship remained significant in participants matched by age, duration of T2DM, SBP, and DBP (*p* < 0.001), demonstrating the robustness of the finding.

## 4. Discussion

This study investigated the association between SAF, an indicator of skin AGE accumulation, and DKD prevalence in T2DM patients, emphasizing SAF as a better biomarker for DKD risk evaluation than HbA1c. The association between SAF and DKD prevalence was independent of several confounders. Moreover, we featured the association in detail, uncovering a dose-response correlation between SAF levels and the odds of developing DKD, both before and after adjusting for potential confounders. Notably, this study is the first to establish linear relationships between SAF and renal function parameters (eGFR and log-transformed UACR) in T2DM patients. Our findings strongly suggest that SAF level is a promising biomarker for identifying T2DM patients with impaired renal function and an elevated risk of DKD.

It has long been the consensus that HbA1c < 7% represents good glycemic control and is recommended as the target for non-pregnant adult T2DM patients [18]. However, HbA1c primarily reflects average blood sugar levels over the preceding 6–8 weeks and does not fully capture the long-term effects of hyperglycemia on body tissues [19]. By contrast, AGEs exhibit better stability and represent cumulative tissue glucose exposure over the past several years, making them more reliable predictors of diabetes-related complications than HbA1c [20,21]. Multiple AGEs possess fluorescence properties. Therefore, total fluorescent AGEs can be measured through the noninvasive technique of SAF [22]. Validation studies have consistently provided evidence of a significant association between SAF and AGE content in skin biopsies. Although SAF assessment may not exclusively represent skin AGEs content due to the presence of other endogenous fluorophores in the skin that emit fluorescence signals similar to skin AGEs, substantial evidence suggests that 76% of the variance in the SAF signal is attributable to fluorescent skin AGEs content [23,24,25]. Furthermore, the correlation between SAF and diabetic complications, both microvascular and macrovascular, has been well-supported by a number of studies [26]. In our study, average HbA1c levels between the DKD group and the non-DKD group showed no significant difference at all, indicating that HbA1c has extremely limited value in predicting the risk of DKD in patients with T2DM. On the contrary, each 1 AU increase in SAF corresponded to a 1.6% rise in DKD prevalence. These results highlight the potential of SAF as a superior biomarker for DKD in patients with T2DM compared to HbA1c.

Previous studies have linked skin AGEs and SAF to kidney disease in patients with and without diabetes [9,27,28]. For instance, data from the Hong Kong Diabetes Biobank revealed that a 30% decline in eGFR occurred more frequently with increasing SAF quartiles [29]. In a cohort comprising well-controlled T2DM patients, SAF level was found to correlate with microalbuminuria development over a 3-year follow-up period [30]. However, existing studies have yet to thoroughly quantify the relationship of SAF with the deterioration of renal function and the progression of kidney disease in T2D, particularly by regarding both eGFR and UACR as continuous variables. Treating eGFR and UACR as continuous variables allows for a more specific and quantitative representation of the relationship between SAF and renal function. Through such an approach, we managed to capture subtle variations in renal function and offered a more precise depiction of the connection between SAF levels and renal function deterioration. In our study, SAF was linearly associated with eGFR and log-transformed ACR in patients with T2DM. For every 1 AU increase in SAF, the eGFR of T2DM patients decreased by 0.14 mL/min/1.73 m^2^, and UACR increased by 1.2%. We thereby provided meaningful data to support the clinical utility of SAF in assessing disease progression and renal impairment in T2DM patients.

The strengths of this study include, firstly, the large sample size and comprehensive clinical data. Secondly, we utilized a validated, noninvasive method to detect and quantify the accumulation of AGEs in tissues. Additionally, we carefully adjusted for potential confounders throughout the whole analysis. All these factors contribute to the reliability of our findings. However, several limitations should be noted. For starters, this is a cross-sectional study, so we were unable to establish a causal relationship between AGEs and the development of DKD. Furthermore, the participants in this study were hospitalized Chinese T2DM patients, who tend to have relatively poor glycemic control, and since racial differences have been known to affect the measurement of SAF [31], whether our findings can be generalized to other T2DM patients requires further investigation. Additionally, our study measured HbA1c via HPLC, which, although widely considered a standard and reliable method, may have limitations in patients with advanced renal dysfunction. Uremic conditions in severe DKD (G5 stage) can lead to altered hemoglobin metabolism, potentially affecting the accuracy of HbA1c measurement. However, it is important to note that most sources of interference have been effectively reduced through improved analytical methodologies [32]. In addition, only three patients in our cohort were classified as G5, limiting the extent to which this issue might have influenced our overall findings.

In conclusion, this study demonstrates that SAF has greater potential than HbA1c for evaluating the risk of DKD in patients with T2DM. The level of SAF is significantly associated with not only the development of the microvascular complication DKD but also the deterioration of renal function in T2DM patients. These findings suggest that SAF is of great potential in the clinical application of DKD screening and risk stratification.

## Figures and Tables

**Table 1:** Baseline characteristics of all participants.

Variable	Without DKD	With DKD	*p* Value ^a^	A1	A2	A3	*p* Value ^b^	G1 and G2	G3	G4 and G5	*p* Value ^b^
No. of Participants	682	577		726	360	173		1119	114	26	
Age, years	68.00 [58.25~74.00]	68.00 [60.00~76.00]	0.025	68.00 [59.00~75.00]	68.00 [59.00~76.00]	68.00 [60.00~75.00]	0.538	68.00 [59.00~75.00]	71.50 [65.00~79.00] ^###^	72.50 [61.25~78.25]	<0.001
Male gender, n (%)	370 (54.25%)	316 (54.77%)	0.900	391 (53.86%)	193 (53.61%)	102 (58.96%)	0.444	506 (45.22%)	54 (47.37%)	13 (50%)	0.815
BMI, kg/m^2^	25.16 ± 11.17	25.17 ± 4.27	0.979	25.2 ± 10.86	24.86 ± 4.42	25.63 ± 4.09	0.621	25.05 ± 9.14	26.14 ± 3.78	25.78 ± 3.89	0.417
SBP, mmHg	134.47 ± 20.54	132.40 ± 20.28	0.150 0.074	134.05 ± 20.29	132.24 ± 20.99	134 ± 19.86	0.442 0.369	133.84 ± 20.38	130.48 ± 20.6	133.19 ± 21.66	0.165 0.246
DBP, mmHg	76.38 ± 11.12	75.48 ± 10.82		76.18 ± 11.04	76.01 ± 11.23	74.99 ± 10.22		76.17 ± 11	74.44 ± 10.97	73.81 ± 10.2	
Duration, years	8.00 [1.00~15.00]	10.00 [4.00~18.00]	<0.001	8.00 [1.00~15.00]	10.00 [3.00~16.00] *	11.00 [8.00~20.00] ***	<0.001	10.00 [2.00~15.00]	10.00 [5.50~20.00] ^#^	20.00 [10.00~20.00] ^###^	<0.001
HbA1c, %	10.24 ± 2.71	10.36 ± 2.86	0.414	10.28 ± 2.74	10.37 ± 2.81	10.22 ± 2.88	0.823	10.33 ± 2.75	10.24 ± 3.06	8.82 ± 2.41 *	0.023
FPG, mmol/L	7.12 [5.96~9.15]	7.36 [5.97~9.68]	0.121	7.12 [5.96~9.17]	7.35 [6.02~9.62]	7.57 [5.88~9.87]	0.356	7.27 [6.00~9.34]	7.12 [5.96~9.65]	6.90 [5.08~9.71]	0.244
FCP, ng/mL	1.42 [0.89~2.08]	1.64 [0.96~2.64]	<0.001	1.48 [0.92~2.15]	1.48 [0.86~2.47]	1.93 [1.14~2.91] ***	<0.001	1.42 [0.90~2.17]	2.45 [1.68~3.39] ^###^	2.73 [1.32~4.94] ^###^	<0.001
GA, %	30.86 ± 12.39	31.30 ± 13.66	0.552	31.04 ± 12.68	31.28 ± 13.01	30.69 ± 14.21	0.885	31.14 ± 12.8	31.6 ± 14.73	25.1 ± 11.72 ^#^	0.040
TC, mmol/L	4.04 ± 0.87	4.08 ± 1.03	0.444	4.03 ± 0.88	4.07 ± 0.93	4.13 ± 1.21	0.491	4.06 ± 0.92	3.98 ± 1.15	4.19 ± 1.18	0.685
TG, mmol/L	1.21 [0.87 ~ 1.74]	1.33 [0.93~1.86]	0.004	1.22 [0.87~1.76]	1.28 [0.91~1.87]	1.35 [0.95~1.78]	0.065	1.22 [0.88~1.75]	1.57 [1.15~2.00] ^###^	1.40 [1.11~2.02]	<0.001
HDL-C, mmol/L	1.11 [0.91~1.32]	1.02 [0.87~1.27]	0.001	1.10 [0.90~1.31]	1.05 [0.89~1.29]	0.99 [0.84~1.27] *	0.019	1.09 [0.90~1.31]	0.94 [0.80~1.18] ^###^	0.87 [0.78~1.32]	<0.001
LDL-C, mmol/L	2.49 ± 0.84	2.51 ± 0.90	0.593	2.48 ± 0.85	2.5 ± 0.8	2.57 ± 1.05	0.602	2.5 ± 0.83	2.45 ± 1.09	2.55 ± 1.11	0.865
eGFR, ml/min/1.73 m^2^	115.65 ± 35.49	93.29 ± 45.25	<0.001	111.71 ± 37.81	107.61 ± 45.82	74.34 ± 34.65 ***	<0.001	113.16 ± 37.42	48.22 ± 8.29 ^###^	22.32 ± 5.39 ^###^	<0.001
UACR, mg/g	8.55 [4.90~14.79]	102.07 [42.45~395.72]	<0.001	8.51 [4.90~14.86]	72.47 [42.04~113.26] ***	856.32 [430.17~1859.10] ***	<0.001	17.10 [7.32~70.22]	53.69 [17.37~731.89] ^###^	1859.10 [977.96~4095.67] ^###^	<0.001
SAF, AU	88.65 ± 15.86	94.13 ± 17.08	<0.001	89.01 ± 15.85	92.85 ± 16.72 ***	96.7 ± 18.1 ***	<0.001	90.31 ± 16.48	97.53 ± 16.7 ^###^	100.12 ± 15.5 ^##^	<0.001
SUA, μmol/L	265.93 ± 78.36	300.71 ± 95.57	<0.001	272.16 ± 83.83	283.32 ± 88.11	319.6 ± 97.14 ***	<0.001	270.07 ± 80.42	372.65 ± 92.45 ^###^	391.92 ± 92.31 ^###^	<0.001
Diabetes family history, n (%)	197 (28.89%)	191 (33.1%)	0.120	205 (28.24%)	125 (34.72%)	58 (33.53%)	0.066	768 (68.63%)	84 (73.68%)	19 (73.08%)	0.490
Hypertension history, n (%)	404 (59.24%)	379 (65.68%)	0.022	441 (60.74%)	231 (64.17%)	111 (64.16%)	0.465	441 (39.41%)	25 (21.93%) ^#^	10 (38.46%)	0.001
Current smoker, n (%)	184 (26.98%)	137 (23.74%)	0.212	194 (26.72%)	91 (25.28%)	36 (20.81%)	0.275	822 (73.46%)	93 (81.58%)	23 (88.46%)	0.043
Medication, n (%)											
Lipid-lowering agents	446 (65.4%)	396 (68.63%)	0.248	476 (65.56%)	243 (67.5%)	123 (71.1%)	0.364	378 (33.78%)	34 (29.82%)	5 (19.23%)	0.218
Aspirin	190 (27.86%)	172 (29.81%)	0.484	204 (28.1%)	108 (30%)	50 (28.9%)	0.808	803 (71.76%)	76 (66.67%)	18 (69.23%)	0.506
Antihypertensive agents	329 (48.24%)	368 (63.78%)	<0.001	363 (50%)	208 (57.78%)	126 (72.83%) ***	<0.001	540 (48.26%)	21 (18.42%) ^###^	1 (3.85%) ^###^	<0.001
Insulin injection	201 (29.47%)	204 (35.36%)	0.030	218 (30.03%)	123 (34.17%)	64 (36.99%)	0.133	783 (69.97%)	62 (54.39%) ^##^	9 (34.62%) ^###^	<0.001
Oral anti-diabetes drugs	488 (71.55%)	393 (68.11%)	0.205	515 (70.94%)	255 (70.83%)	111 (64.16%)	0.199	327 (29.22%)	36 (31.58%)	15 (57.69%) ^#^	0.007

Data are expressed as mean ± SD, median (interquartile range), or n (%). Abbreviation: SBP: systolic blood pressure; DBP: diastolic blood pressure; BMI: body mass index; HbA1c: glycated hemoglobin A1c; GA: glycated albumin; FPG: fasting plasma glucose; FCP: fasting C-peptide; TC: total cholesterol; TG: triglyceride; HDL-C: high-density lipoprotein Cholesterol; LDL-C: low-density lipoprotein Cholesterol; EGFR: epidermal growth factor receptor; UACR: Urinary albumin creatinine ratio; SAF: Skin autofluorescence; SUA: Serum uric acid. ^a^ independent samples *t*-test, Mann–Whitney Wilcoxon test, or Chi-square test. ^b^ ANOVA tests, Mann–Whitney Wilcoxon tests, or Chi-square tests. * *p* < 0.05, *** *p* < 0.001, A1 vs. A2 or A3. ^#^ *p* < 0.05, ^##^ *p* < 0.01, ^###^ *p* < 0.001, G1 and G2 vs. G3 or G4 and G5.

**Table 2:** Baseline characteristics of matched participants.

Variable	Without DKD	With DKD	Statistics Value	*p* Value ^a^
No. of Participants	563	563		
Age, years	69.00 [60.00~75.00]	68.00 [60.00~76.00]	−0.116	0.907
Duration, years	10.00 [3.00~16.00]	10.00 [4.00~17.00]	−1.311	0.190
HbA1c, %	10.29 ± 2.69	10.31 ± 2.84	−0.141	0.888
SBP, mmHg	133.27 ± 20.52	132.59 ± 20.25	0.560	0.576
DBP, mmHg	75.85 ± 11.02	75.66 ± 10.82	0.292	0.770
Male gender, n (%)	293 (52.04%)	308 (54.71%)	0.700	0.403
BMI, kg/m^2^	25.18 ± 12.17	25.17 ± 4.30	0.022	0.983
FPG, mmol/L	7.18 [6.00~9.25]	7.35 [5.96~9.61]	−0.781	0.435
C-peptide, ng/mL	1.41 [0.88~2.06]	1.65 [0.97~2.64]	−4.289	<0.001
GA, %	31.05 ± 12.34	31.08 ± 13.65	−0.044	0.965
TC, mmol/L	4.01 ± 0.90	4.08 ± 1.03	−1.202	0.229
TG, mmol/L	1.20 [0.86~1.74]	1.33 [0.94~1.86]	−2.982	0.003
HDL-C, mmol/L	1.12 [0.90~1.33]	1.02 [0.87~1.27]	−3.263	0.001
LDL-C, mmol/L	2.45 ± 0.85	2.52 ± 0.90	−1.347	0.178
eGFR, mL/min/1.73 m^2^	114.00 ± 34.17	93.35 ± 45.18	8.645	<0.001
UACR, mg/g	8.83 [5.03~15.52]	100.77 [42.32~392.49]	−26.925	<0.001
SAF, AU	89.58 ± 15.89	93.97 ± 17.14	−4.455	<0.001
SUA, μmol/L	263.85 ± 79.67	300.63 ± 95.65	−7.011	<0.001
Diabetes family history, n (%)	153 (27.18%)	187 (33.21%)	4.590	0.032
Hypertension history, n (%)	338 (60.04%)	370 (65.72%)	3.660	0.056
Current smoker, n (%)	145 (25.75%)	132 (23.45%)	0.690	0.406
Medication, n (%)				
Lipid-lowering agents	383 (68.03%)	387 (68.74%)	0.040	0.848
Aspirin	169 (30.02%)	169 (30.02%)	0.000	1.000
Antihypertensive agents	289 (51.33%)	357 (63.41%)	16.300	<0.001
Insulin injection	177 (31.44%)	196 (34.81%)	1.300	0.254
Oral anti-diabetes drugs	408 (72.47%)	386 (68.56%)	1.880	0.170

Data are expressed as mean ± SD, median (interquartile range), or n (%). ^a^ independent samples *t*-tests, Mann–Whitney Wilcoxon tests, or Chi-square tests.

**Table 3:** Logistic Regression Analysis Evaluating DKD as Dependent Variable.

Independent Variables	Simple Regression				Stepwise Multiple Regression				
	Estimate	Standard Error	*Z* Value	*p* Value	Estimate	Standard Error	*Z* Value	*p* Value	OR (95%CI)
Age	0.010	0.004	2.318	0.020					
Duration	0.031	0.007	4.393	<0.001	0.025	0.007	3.364	0.001	1.03 (1.01–1.04)
C-peptide	0.248	0.049	5.024	<0.001	0.171	0.054	3.185	0.001	1.19 (1.07–1.32)
TG	0.117	0.056	2.077	0.038					
HDL-C	−0.539	0.175	−3.069	0.002	−0.278	0.188	−1.482	0.138	0.76 (0.52–1.09)
SAF	0.020	0.004	5.726	<0.001	0.016	0.004	4.278	<0.001	1.02 (1.01–1.02)
SUA	0.005	0.001	6.800	<0.001	0.004	0.001	5.034	<0.001	1 (1–1.01)
Hypertension history	0.275	0.117	2.348	0.019					

Abbreviations: OR: odds ratio; CI: confidence interval.

**Table 4:** Linear Regression Analysis Evaluating eGFR as Dependent Variable.

Independent Variables	Estimate	Standard Error	Statistics	*p* Value
Age	−1.135	0.077	−14.809	<0.001
C-peptide	−6.351	0.788	−8.061	<0.001
SAF	−0.140	0.060	−2.324	0.020
SUA	−0.157	0.011	−14.157	<0.001
Hypertension history	−5.227	2.002	−2.611	0.009
*F* value		148.670		
*F*-value-associated *p* value		<0.001		

**Table 5:** Linear Regression Analysis Evaluating ln(ACR) as Dependent Variable.

Independent Variables	Estimate	Standard Error	Statistics	*p* Value
Duration	0.038	0.006	5.984	<0.001
C-peptide	0.167	0.042	3.990	<0.001
SAF	0.012	0.003	3.952	<0.001
SUA	0.003	0.001	4.265	<0.001
*F* value		30.111		
*F*-value-associated *p* value		<0.001		

## Data Availability

Some or all datasets generated during and/or analyzed during the current study are not publicly available but are available from the corresponding author upon reasonable request.
